# Automatic detection of fish scale circuli using deep learning

**DOI:** 10.1093/biomethods/bpae056

**Published:** 2024-07-31

**Authors:** Nora N Hanson, James P Ounsley, Jason Henry, Kasim Terzić, Bruno Caneco

**Affiliations:** Freshwater Fisheries Laboratory, Marine Directorate, Scottish Government, Pitlochry PH16 5LB, United Kingdom; Freshwater Fisheries Laboratory, Marine Directorate, Scottish Government, Pitlochry PH16 5LB, United Kingdom; Freshwater Fisheries Laboratory, Marine Directorate, Scottish Government, Pitlochry PH16 5LB, United Kingdom; School of Computer Science, University of St Andrews, St Andrews KY16 9SX, United Kingdom; Freshwater Fisheries Laboratory, Marine Directorate, Scottish Government, Pitlochry PH16 5LB, United Kingdom

**Keywords:** scale, Convolutional Neural Network, circuli, growth, deep learning, salmon

## Abstract

Teleost fish scales form distinct growth rings deposited in proportion to somatic growth in length, and are routinely used in fish ageing and growth analyses. Extraction of incremental growth data from scales is labour intensive. We present a fully automated method to retrieve this data from fish scale images using Convolutional Neural Networks (CNNs). Our pipeline of two CNNs automatically detects the centre of the scale and individual growth rings (circuli) along multiple radial transect emanating from the centre. The focus detector was trained on 725 scale images and achieved an average precision of 99%; the circuli detector was trained on 40 678 circuli annotations and achieved an average precision of 95.1%. Circuli detections were made with less confidence in the freshwater zone of the scale image where the growth bands are most narrowly spaced. However, the performance of the circuli detector was similar to that of another human labeller, highlighting the inherent ambiguity of the labelling process. The system predicts the location of scale growth rings rapidly and with high accuracy, enabling the calculation of spacings and thereby growth inferences from salmon scales. The success of our method suggests its potential for expansion to other species.

## Introduction

Longitudinal measurement of individual growth history has become an essential tool for understanding variability in successful life histories of wild salmonids, and the environmental factors which influence them. Such growth histories may be retrieved from individual fish that have been repeatedly caught and measured (a technically difficult and costly sampling process for wild fish), or inferred from patterns of incremental scale growth which is manifest in teleost fish as the formation of distinct growth rings, or circuli, in the calcified part of the scale [1]. In salmon, somatic growth in length is demonstrably correlated with scale growth [2–4] and, because they are routinely used in ageing salmon, many multi-decadal scale archives exist. Consequently, growth data derived from scales has become extremely useful to evaluate the effects of climate on salmon growth, survival and life history characteristics [5–8].

Extraction of detailed salmon scale growth increment data has traditionally been conducted by experienced scale readers. These experts use image analysis tools to aid in recording growth increments along a single axis, while also identifying features of interest based on specific patterns in scale growth [6–9]. All individual circuli may be demarcated [6], or analyses may focus on a defined set of growth features such as transition to freshwater, growth maxima or minima [7, 9]. Demarcating individual circuli, in particular, is time consuming when a single scale may present hundreds of circuli. Motivated by similar challenges in age determination from other calcified structures (otoliths, fin rays, bones), various computer-assisted age and growth estimation systems have been developed to aid in the demarcation of growth features [10]. The most commonly cited systems within salmon scale growth increment analyses are proprietary software ImagePro from MeidaCybernetics, and plugins available as open-source software. These tools are semi-automated 1D or 2D pattern recognition systems that detect circuli along a user-defined scale image transect based on pixel intensity profiles, but these require interactive correction, insertion, or deletion of individual circuli. More recently, Chaput and Chaput [11] developed a semi-automated approach to extracting scale growth information based on spectral analysis which requires the specification of the scale centre before pixel saturation along radial transects are extracted.

Given the extensive image banks of fish scales maintained by many fisheries research bodies for fish ageing purposes, the development of fully automated, algorithm-based tools to streamline the extraction of growth increments data from these images is highly desirable. Such advancements would enable the creation of historical catalogues of high-fidelity growth data. Recently, fully automated deep learning techniques, and especially Convolutional Neural Networks (CNNs), have been applied to age and life history estimation challenges in fisheries research using images of whole scales or otoliths [12, 13]. However, applications developed to date tend to focus on the determination of specific features (e.g. age, spawner/non-spawner, wild/farmed) and do not include growth increment extraction or circuli demarcation.

This study explored the automated extraction of features from Atlantic salmon scale images utilizing CNNs. The aim was to develop a tool to automatically delimit the centre of a salmon scale and the subsequent location of individual circuli bands along multiple transects emanating from the centre of the scale to its edge, from which circuli spacings can be derived. By considering multi-pixel radial transects from images of scale impressions, the extraction of circuli spacings can be re-framed as an object detection problem, which is well-studied in computer vision and machine learning research [14]. Recent advancements in deep learning and specifically CNNs [15–17] have led to the development of numerous tools which are highly proficient at locating objects within images. CNNs are designed to automatically learn hierarchical patterns and features from input images by mapping those features to annotated target objects through supervised learning [18]. Once appropriately trained, CNNs can accurately detect and classify target objects in new images.

Here, we present an approach that leverages the existing object detection algorithm YOLOv3 [19] to automate the identification of circuli positions within individually defined bounding boxes along a scale image transect from which circuli spacings, and hence of growth increment data, can be extracted.

## Materials and methods

**Figure 1.**
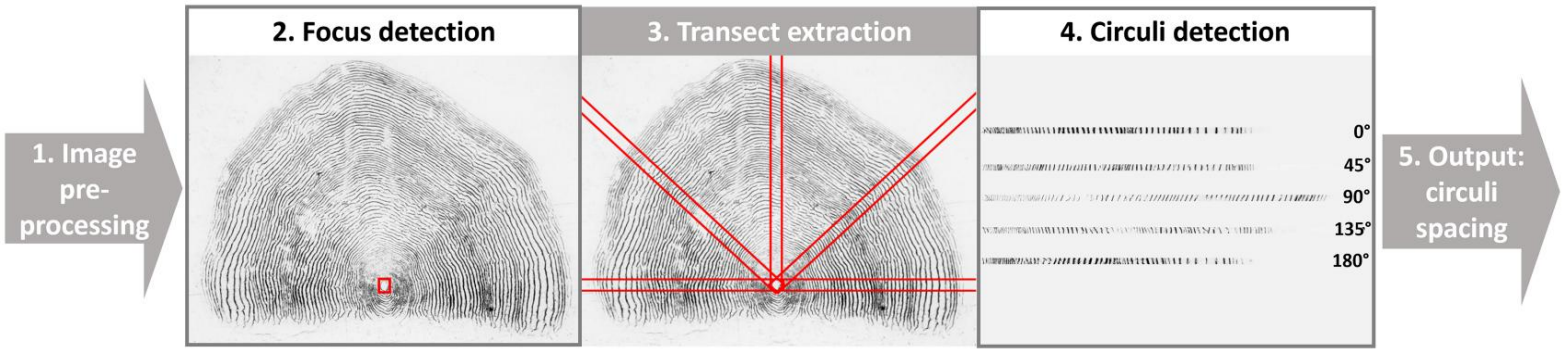
Conceptual diagram of circuli detection pipeline: 1. Preprocessing of images; 2. processed images are run through a focus detector which returns a single bounding box around the focus; 3. focus bounding boxes are used to extract radial transects from the original image at configurable angles; 4. each transect image is run through a circuli detector which returns a bounding box for each predicted circulus along the transect; 5. spacings calculated as distances between ciculi bounding box centres are returned to the user

Automated circuli spacing extraction was achieved through a sequence of image processing and object detection steps (Fig. 1). This pipeline takes scale images as input and outputs the absolute distances (in pixels) between centres of bounding boxes prescribing circuli along radial transects originating at the scale focus. Central to the pipeline are two object detectors: a focus detector and a circuli detector.

### Data preparation

A repository of 1035 salmon scales was available to train object detectors (1022 adults and 13 smolts from a representative range of age classes for wild Atlantic salmon returning to the east coast of Scotland). High resolution images (3840 × 2748 pixels) were captured from acetate impressions of each scale using a compound microscope (Olympus BX43) fitted with a digital colour camera (Olympus SC100). The eyepiece optics were 10× to the camera. Using a 0.5× camera adapter and an 1.25×/0.04 objective, the total magnification was 6.25× to the camera. Each scale was imaged with the longest radial axis oriented vertically.

Focus annotations were created by delimiting bounding boxes around the entire focus of each scale image using the *LabelImg* package [20] (Fig. 1—step 2). Focus bounding boxes were used to derive radial transect images from each scale image. The centre of the focus bounding box determined the centre of the radial transect with a width equal to half the width of the smallest bounding box edge and angle equal to that specified by the user. Thus, all transects (of any angle) from an individual scale image had the same width, and transect length varied by angle. Circuli annotations were then delimited for each circulus along a randomly sampled transect angle via the same labelling method as the foci. This process resulted in a total of 1035 individual scale foci and 58 023 circuli annotations belonging to 813 radial transects extracted randomly at angles of 0, 45, 90, 135 or 180 degrees relative to the horizonal axis of the scale image, pivoting at the centre position of the identified focus (Fig. 1—step 3). Scales chosen for transect extraction were selected randomly from the available pool. The angles were chosen as a subset representative of the variation in circuli deposition seen across a typical salmon scale (Fig. 1). For both detectors, the images (whole scale and transects) were partitioned into training, validation, and test sets with a 70:20:10 split, respectively. A single labeller created all annotations, which were considered the ‘ground truth’ for training and evaluation purposes. We comment on the implications of this in the discussion.

### CNN training and performance evaluation

The two object detectors use YOLOv3; a popular CNN object detection algorithm as of 2018 [16, 19, 21]. YOLOv3 employs a regression approach to efficiently position bounding boxes around objects at multiple scales, achieving a good trade-off between detection accuracy and training speed on common object detection problems [19]. Training for both detectors was performed via transfer learning, where the feature-extraction layers (Darknet-53) of the YOLOv3 model were initialized with publicly available weights from a model pre-trained on the ImageNet dataset [22]. Throughout training, the Darknet-53 layers remained frozen, while the top detection layers were trainable.

All code was implemented in Python version 3.7, utilizing a Tensorflow/Keras implementation of the YOLOv3 model developed by Zhang *et al*. [23]. Tensorflow version 2.1.0 [24] and Keras version 1.1 [25] were used for this purpose. Training was conducted using an 8GB NVIDIA GeForce GTX 1080 GPU card geared with CUDA v10.1 for GPU-accelerated parallel computations [26].

The CNN settings used for training and performance evaluation of each detector are outlined in Table 1. Based on batch size, set to ensure efficient training iterations while minimizing memory requirements, the input resolution was maximized with respect to the available RAM. Input resolutions were set to the maximum values observed in the training dataset and the original YOLOv3 code was adapted to support rectangular input images for the circuli detector as preliminary results indicated that square inputs had a notable impact on the performance of the circuli detector. A somewhat arbitrary number of epochs was set to ensure training convergence, with early stopping employed to prevent overfitting; this mechanism automatically halts training when the validation error reaches a minimum at the end of an epoch. Training used the Adam optimization algorithm [27], with the learning rate set to the default value specified in the YOLOv3 implementation. Additionally, for the circuli detector, the Keras option of performance-based learning scheduling was utilized to reduce the learning rate when training progress stagnated [28]. Minimum YOLOv3 confidence scores of 0.5 and 0.3 for, respectively, the focus and circuli detector were required when accepting detections. Performance evaluation of each object detector was based on intersection over union (IOU) threshold of 0.5 between detections and ground truths bounding boxes.

**Table 1.** CNN settings used for training and evaluating the focus and circuli detectors.

CNN settings	Focus detector	Circuli detector
Input image resolution [height × width]	[1376 × 1376]	[64 × 3904]
Batch size	6	8
Number of epochs (early-stop patience)	250 (8)	250 (8)
Learning rate	0.001	0.001
Learning schedule factor (patience)	–	0.66 (3)
YOLO score threshold	0.5	0.3
IOU threshold	0.5	0.5

Input resolution determines the dimensions (in pixels) of the YOLOv3’s input layer; batch size defines the number of images processed per training iteration; epoch denotes a complete pass of the training dataset through the algorithm (i.e. over all batches), with early-stop patience setting the number of epochs without improvements before training is halted. Learning rate determines the step size taken at each iteration of the optimization algorithm to update the model’s parameters, affecting how quickly a model converges; learning schedule factor and patience determine, respectively, the reduction in learning rate and the number of epochs without improvements before learning rate is reduced; YOLO confidence scores encapsulate both the estimated probability that the target object appears in the detection box and how well the box fits the object [16]; IOU quantifies the overlapping area between detection and ground truth bounding boxes, normalized by their union area—IOU threshold defines the value above which detections are deemed correct (i.e. true positives).

Performance of both detectors was assessed against the test set using average precision (AP) which combines two standard evaluation metrics [29]: precision and recall. Precision is the proportion of correct detections (number of correct detections divided by the total number of detections):

$$\frac{\mathrm{TP}}{\mathrm{TP}+\mathrm{FP}}$$

Recall is the proportion of correctly detected growth truths (number of correct detections divided by the total number of ground truth objects):

$$\frac{\mathrm{TP}}{\mathrm{TP}+\mathrm{FN}}$$

where TP is ‘true positive’, or the correct detection of a circuli at the same location as the ground truth bounding box (i.e. IOU ≥ 0.5). FP is ‘false positive’, or an incorrect detection of a circuli where there was no ground truth bounding box (IOU < 0.5). FN is ‘false negative’, or a missed detection of a ground truth bounding box.

AP is the area under the precision-recall curve and was calculated using the toolkit developed by Padilla *et al*. [29]. Differences in precision and recall among transects originating from different angles within the test set were tested using Kruskal–Wallis rank sum tests.

In addition, a second set of circuli annotations for 87 transects (0 and 90 degree angles, from images collected in the same way) was created by an independent human labeller as means of assessing inter-labeller error. The precision, recall and F1 scores (harmonic mean of precision and recall) [28] of both the circuli detector and the human labeller against the ground truth for these transects were used for comparison. Additionally, we calculated the average number of circuli missed (false negative) or added (false positive) and the average percentage difference in the total number of circuli counted on each transect by the circuli detector or the second human labeller. The accuracy of detected circuli positions was evaluated by calculating the Mean Center Error (MCE), which is the Euclidean distance (in pixels) between the centres of the detected and ground truth bounding boxes, averaged over the transect. Note that all outputs were on the scale of the original image (not influenced by the input resolution setting of the CNN).

## Results and discussion

### CNN evaluation

The focus detection CNN achieved 99% AP on the test set, with 102 foci correctly detected, one focus missed and none incorrectly detected. On examination of validation and test set images where focus detection failed it was clear that poor scale and/or image quality were responsible. It was critical for the focus detection step that scales with damaged or incomplete centres are excluded and that care is taken to prepare a clear image free of debris. The circuli detection CNN achieved 95.15% AP on the test set, with 5827 circuli correctly detected, 221 missed, and 549 incorrectly detected (from a total of 81 transects; Table 2). There were no significant differences in precision or recall among transects from different positions (angles) on the scale images (Kruskal–Wallis test, *P *=* *.11). The focus detection CNN processed 100 images in seconds and the circuli detector CNN processed 100 images within minutes; both processes can be sped up by restricting optional diagnostic outputs. For context, it may take a human analyst 10–15 min to process a single transect on a single scale, using existing pixel saturation-based software to aid the process.

**Table 2.** Number of objects used in training, validation and testing of two convolutional neural networks, together with mean average precision (AP).

	Training	Validation	Test	AP (%)
Focus detector	725	207	103	99
Circuli detector	40 678 (569)	11 297 (163)	6048 (81)	95.1

Objects were bounding boxes around foci for the focus detector and around circuli for the circuli detector. The number of transect images is provided in parentheses.

### Uncertainty in detections

**Figure 2.**
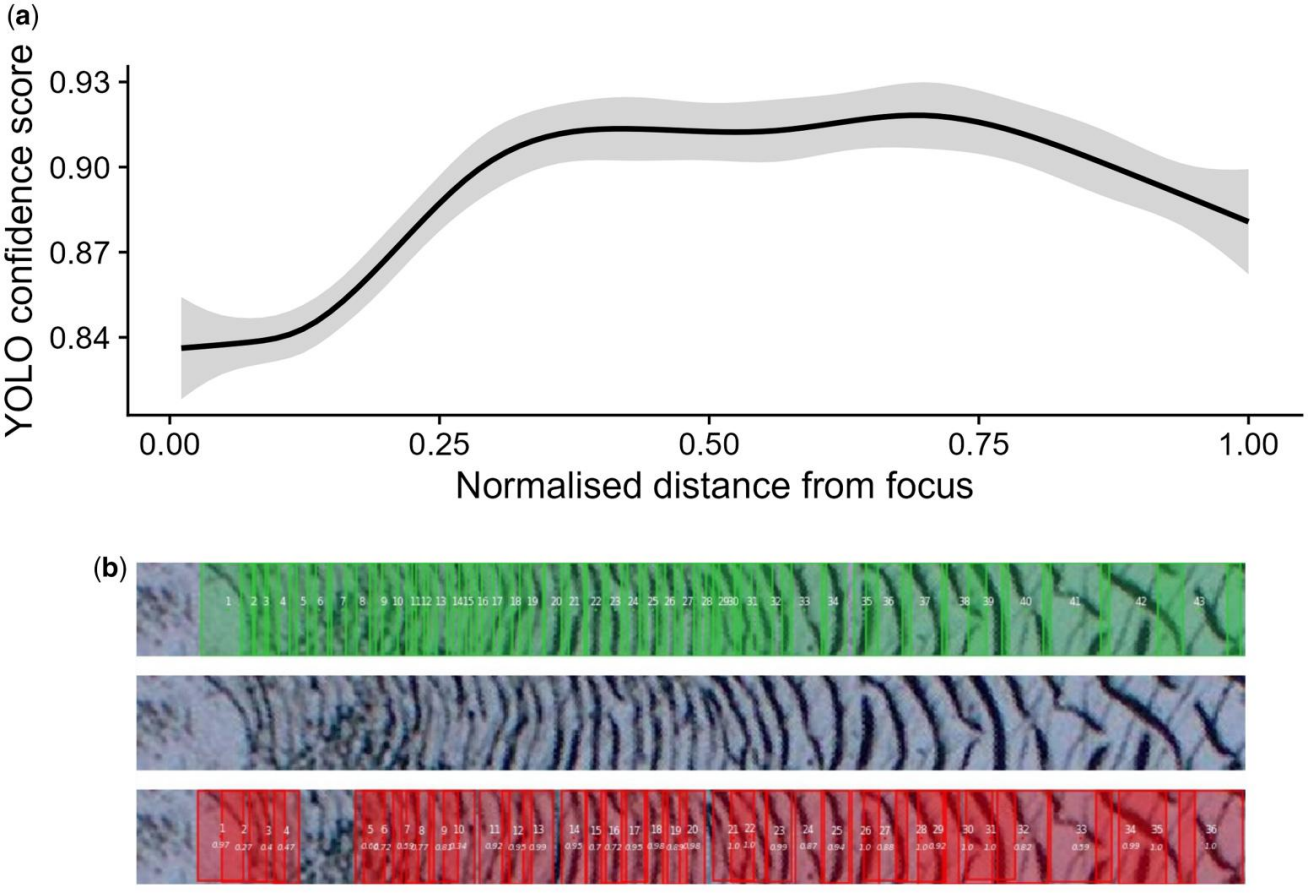
Confidence score of circuli detections (as provided by YOLOv3) as a function of (normalized) distance from the scale focus (a) demonstrating lower confidence in detections within the zone of narrow spacings during juvenile growth. Transect images superimposed with ground truth detections (green boxes) and CNN detections (red boxes) provide a useful visualization to further evaluate errors (b)

Further inspection of the circuli detection outputs showed a clear pattern of decreased detector confidence for circuli within the freshwater (juvenile) zone of the salmon scale image (Fig. 2a). Given the physical structure of adult salmon scales, the freshwater zone comprises a smaller proportion of the whole scale than does the marine zone and circuli within. Within this region, circuli spacings are narrowest and individual circuli are often difficult to discern. Figure 2b provides an illustrative example.

The independent human labeller slightly outperformed the circuli detector, though results were comparable (Table 3). Compared to a second labeller, the circuli detector was slightly more likely to incorrectly identify a feature as a circulus (lower precision) than to fail to detect a circulus (similar recall). The circuli detector and the second labeller performed similarly in terms of the number of circuli detected relative to the ground truth: 3.4% (median; −5.4%, 15.1%, 5th and 95th quantile) difference for the circuli detector versus a 3.4% (median; −1.7%, 16.6%, 5th and 95th quantile) difference for the second human labeller. Similarly, transect-level MCE was comparable at a median of 1.35 px (range 0.75–4.1) for the circiuli detector and a median of 0.71 px (range 0.37–3.9) for the second human labeller. This comparison highlights the inherent ambiguity in the ‘ground truth’ as created by a single labeller. The disagreement between the circuli detector and the ground truth was similar to that which would be expected between two human labellers.

**Table 3.** Total number of correct detections (true positives; TP), incorrect detections (false positives; FP), missed detections (false negatives; FN), and the average precision, recall and F1 of the circuli detector and of a second labeller when compared to labeller one (ground truth) across 87 transects.

	Circuli detector	Human labeller
TP	5944 (66; 47–94)	6060 (66; 49–100)
FP	617 (5; 1–16)	546 (4; 0–16)
FN	375 (2; 0–13)	259 (1; 0–12)
Precision	0.90 (0.93; 0.78–0.98)	0.95 (0.95; 0.77–1)
Recall	0.94 (0.97; 0.84–1)	0.96 (0.98; 0.89–1)
F1	0.92 (0.94; 0.82–0.98)	0.94 (0.96; 0.84–0.99)

Median values of TP, FP, FN, and F1 per transect are provided in brackets, followed by the 5th and 95th percentile.

Labelling circuli along narrow transect images (Fig. 2b) can be difficult and made more challenging by poor scale impressions and/or image quality. A small area of poor impression, or debris, on a scale image can create sections of what appear to be gaps in circuli transect images. However, detection of features from good quality images will also vary simply due to the nature of scale growth, and human error. Circuli bands may appear broken in places and intersect (‘cut over’) in others [30], likely contributing to variation in spacings among scales from the same individual and among multiple transects taken from the same scale [31].

The supervised learning employed in the present method therefore relies on a ‘ground truth’ that is inherently ambiguous; target objects were marked manually and are subject to human error and labelling ambiguity. This impacts the detector both in training and in evaluation where performance metrics are highly dependent not only on the accuracy of the detector, but also on the quality of annotations used in the evaluation. Labelling inconsistencies among domain ‘experts’ (i.e. labellers) are relatively well recognized within artificial intelligence applications and do not necessarily undermine the method [32, 33]. However, interpretation of evaluation metrics must be considered within the context of ambiguous ‘ground truths’.

Image plots contrasting detections against annotations (as in Fig. 2b) help to scrutinize if any apparent drops in performance metrics are being driven by a deteriorating detector, by poor labelling, or both.

**Figure 3.**
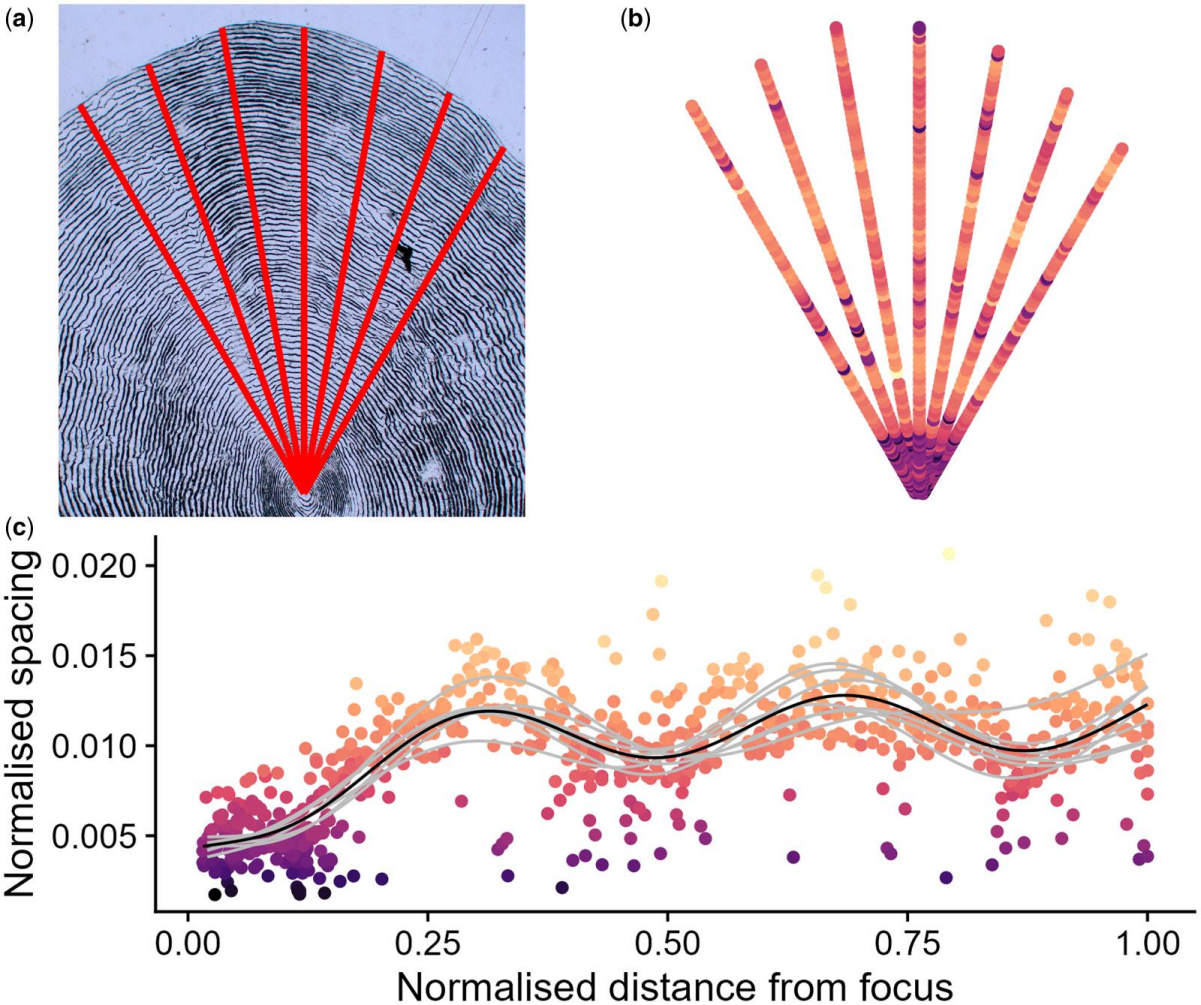
Using the pipeline presented here, (a) multiple radial transects may be extracted from a single scale and (b) inter-circuli spacings extracted and mapped to visualize patterns. (c) Individual transect spacings can be extracted and combined to incorporate variation into a common pattern (method here utilizes hierarchical generalized additive model smooths applied to normalized data). Red lines indicate radial transect paths, dark purple points indicate narrower spacings and lighter yellow indicates wider spacings

At present, inter-labeller or intra-individual variability in ages or growth estimation is rarely, if ever, incorporated into subsequent analyses due to the time investment required to produce data. The two-step detection method presented here allows rapid estimation of within-individual uncertainty as transects can be extracted from multiple scales per individual and at any angle from the scale focus (Fig. 3). As an example application of the CNN detectors developed here, multiple transects may be standardised through simple normalization (division by maximum value observed on the transect) and combined using hierarchical generalized additive model smooths (*mgcv* package); [34]. In doing so, distinct growth seasons (analogous to age in years in this species) can be visualized and predicted with error (Fig. 3c). With testing against new images and potential re-training with annotations from multiple labellers, the algorithm may in fact become less susceptible to individual bias, judgement or error, providing a ‘standardised’ method to extract large volumes of data from existing scale archives. Unacceptable deterioration in performance in the freshwater section of the scale may be ameliorated by inclusion of images at higher magnification, focused on this zone.

## Conclusion

We implemented a deep-learning system to automate the extraction of growth information from digital images of Atlantic salmon scales. There are numerous CNN architectures that one could explore in future work for detecting individual circuli. In this work, we focused on applying a standard off-the-shelf algorithm and found the approach to yield good performance on real scale images. This system can predict the location of scale growth rings rapidly and with high accuracy, enabling the calculation of spacings and thereby growth inferences, with the potential to predict age from distinct growth seasons. Accuracy can be further enhanced with the addition of annotations and re-training the model as required. The entire pipeline for applying the trained CNN to new scale images has been packaged into a user-friendly Python tool, available for use by others with minimal computing resources required (link in Data Availability section)—provided that new scale images are captured under similar conditions to the training data. The information extracted from scale circuli spacings can be used to improve our understanding of the survival of salmon at sea and, with further development, to predict juvenile and adult ages. Whilst annotation of the initial training datasets can be resource intensive, implementation of a machine learning approach to this problem enables more rapid throughput of images than existing techniques, allowing the power of large historical scale archives to be leveraged. The demonstrated success of the developed methodology suggests its potential for expansion to other species, possibly through retraining on new images, with expected similar levels of performance as observed here. However, the generalizability of the current tool to images from other collections or other species must be robustly tested through formal evaluation before being widely applied.

## Data Availability

The code developed here to process scale images is freely available and can be accessed at: https://bitbucket.org/ffl-salmon-at-sea/sscd/src/main/
